# Report on Obstetrics

**Published:** 1869-08

**Authors:** H. B. Buck

**Affiliations:** Chairman of Committee


ARTICLE XXXI.

### REPORT ON OBSTETRICS.

BY H. B. BUCK, M.D., Chairman of Committee.

(Read before the Illinois State Medical Society.)

MR. PRESIDENT AND GENTLEMEN OF THE SOCIETY:

As Chairman of your Committee on Obstetrics, I have the honor to submit the following report:—I shall premise by promising not to weary your patience with speculative theories, but, so far as possible, deal with such material as results from experience and bedside observation; believing that those theories only are valuable which are based upon closely observed and well ascertained facts.

It will not do, perhaps, to declare with Sylvius, "that nothing must be adopted in medicine that has not been confirmed by the senses;" but I do insist that every theory must be rejected that will not stand the test of practical experience.

There was a time when medicine was defined "the art of healing." May we not be justly proud of the profession of to-day, when compared with the primitive ages, when such a definition was as comprehensive as the condition of the science would permit? The tender shoot of that day has waxed stronger and stronger—branch after branch hath put forth, until it hath attained even magnificant proportions.

Since the addition of that important branch—hygiene, or prophylaxy, and one still later, which I will denominate orthopedia, and which in its most extended sense contemplates the correction of all deformities, we catch a glimpse of the extended field for observation, and begin to realise the importance of that science which in these later days has for its object the preservation of health, the cure of disease, and the physical perfection of man.

This definition is sufficiently comprehensive for all time to come—this must ever be the final aim of our noble profession.

It may be said in all ages with equal truth: "*Ars medica*

*est id quod est propter therapeuticen*; therapeutics is the channel through which mankind receive all the beneficent blessings flowing from the science of medicine, whether our efforts are directed to the prevention or cure of disease.

The great question of our lives should be, how can we best prepare ourselves to meet the demands made upon us? I answer that no efforts will more surely redound to our personal advancement than those made for the good of the profession at large.

The keeping of detailed records of cases treated will alone enable us to amass that fund of experimental knowledge necessary for the foundation of even reliable theories—and here I might say, that no addition made has ever stood the test that rested upon other than this as a foundation.

This is the secret of the rapid advance in practical medicine during the past few years; and I verily believe that to achieve even greater successes needs only a combined and more general effort. Of none among the various branches of medicine can this be said more truthfully than the obstetric.

If I lay myself liable to the charge of having made a digression, I can only say, that my object will have been attained if I can so magnify the importance of systematic record keeping, as to induce every member of this Society, in future, to be prepared, when called upon, to add his mite to the general interest of these reports. It is not necessary that some unheard of thing should transpire in order to make an item of interest.

Facts, the accumulation of facts, are what is wanting. It is the multitude of witnesses bearing the same testimony that incontrovertably establish the point.

Your Committee wish to return their grateful acknowledgement to the few among the many invited who have contributed valuable material for this report. The Society will undoubtedly hold them in grateful remembrance.

Several cases of considerable interest have during the past few years fallen under my own observation, bearing upon the causes operating to produce dysmenorrhœa. We meet it among the married and unmarried; and not infrequently find it in the former the result of the real cause of barrenness. These cases might be appropriately termed organic, in contradistinction to those of a nervous or congestive character, where the most minute examination fails to reveal any structural change.

I always act upon the principle that it is better to avoid the use of the speculum until all other appropriate treatment had been tested, especially among the unmarried; but I am quite convinced that many cases depending upon similar causes and amenable to similar treatment endure years of unnecessary misery and medicinal dosing, through feelings of false delicacy, or our dislike even to suggest the proper treatment.

### Case I. dysmenorrhœa.

Mrs. S., aged 21, American, married two years, robust in appearance, and usually enjoyed good health, aside from her menstrual trouble. From the first appearance of her menses, she had suffered intense pain at every period, and had also been subject to great irregularity. The mother stated that when quite young she had several falls, and upon one occasion had fallen astride a fense rail, and had been somewhat injured internally. Was inclined to ascribe all her sufferings to that accident.

I attempted to relieve her sufferings upon several occasions, and was not altogether unsuccessful, but no permanent good was accomplished, other than to correct somewhat the unnatural appearance of the flow. The desire for a family led them to consult me as to the probable cause of delay. Having stated to her previously that an examination might reveal the cause, I found her quite willing to submit to any treatment that might promise success.

Upon examination, I found it difficult to pass Glen's flexible silver probe through what at first appeared a constriction in the cervical canal, upper third. Upon further exploration, I found it to be an exceedingly firm membranous partition, with a small opening at one side. The probe in passing it gave the sensation of extreme firmness.

Ineffectual efforts at passing a dilating instrument occasioned great pain. I concluded to divide it, and then use the necessary means to preserve a free opening.

Using the probe as a director, with a long narrow blade (edge protected to within a short distance of the point), I succeeded in opening the canal, so as to allow the passage of a moderate sized bougie at once. I then introduced a plug of compressed sponge, with a view to dilate the whole canal, with instructions to remove it if very painful. She could tolerate it a few hours only. On the following day, a similar but smaller plug was introduced, to remain for 12 hours. The bougie was passed daily, until the advent of the next period, which came on without pain, and natural in every respect. I have only to add, this was her last period up to date. She is now nursing a fine boy, some 12 months old.

CASE II. DYSMENORRHŒA WITH CONVULSIONS.

Mrs. B., aged 25 years, American. First appearance of menses at the age of 15 years. Had no difficulty the first year: was caught in a shower three days before her period; caused a partial suppression; suffered severe pain. During the next two years she suffered at times, but not at every period. From 18 years of age she had constant trouble; pain increasing in violence, until spasms supervened, in the spring of 1866. I saw her as consulting physician in one of these attacks, in the autumn of 1866. She married a short time after this attack. I learned that during the winter there was no abatement, but rather an increase in the violence of her attacks. She became weak and emaciated; was hardly able to be out of bed. I was called to attend her in June, 1867; was then having a terrific attack. Learning something of her condition, I took along ether, Richardson's vaporizer, speculum, etc. Introducing the speculum I threw the vapor of ether upon the whole surface of the neck of the womb, for a short period, and leaving the speculum in situ, also vaporized the whole hypogastric region. The effect seemed quite satisfactory. I found that the bladder was distended to a considerable extent. Attendants told me that such was always the case.

After the use of the catheter relief seemed complete. A tolerably free use of anti-spasmodic and anodyne remedies prevented further spasms during the attack. She was very soon put upon the following treatment:—

Ammo. Bromidum internally, combined with a vegetable bitter, and the use of the tepid water douche, twice daily; patient in a semi-recumbent posture, using a small tube and such a quantity of water as to occupy 15 or 20 minutes in the operation. This was continued until the next period. Effect—relief to some extent; did not avert the spasm, but patient suffered much less; same retention of urine, but finally passed it without help. After this attack, the same treatment was continued, with the addition of a mild chalybeate tonic; also used the compressed sponge, to dilate the cervical canal, under the impression that although not absolutely constricted, the engorgement consequent upon the menstrual function so narrowed it as to prevent the ready escape of the flow. Results proved the correctness of my suspicion. She had no serious suffering from that time. The same treatment was continued for several months, more attention being given to the restoration of her general health. She was sent to Michigan, among her kindred and friends; and during the few months spent there improved in flesh and strength.

She still used the douche, but less frequently, for a period of at least 10 months. Her general health constantly but slowly improved. The distress which every period occasioned seemed to grow even lighter, *pari passu*, with her general improvement. She is now in excellent health and between five and six months gone in pregnancy.

I might detail other cases of a similar character, but through fear of prolonging this report beyond a proper limit will desist.

### MISCARRIAGE.

It has been a subject of remark among the members of the Sangamon County Medical Society that the present spring has furnished an unusually large number of these cases. One member stated that an Eastern correspondent had called attention to the fact that the same was true of that locality.

How generally it applies, I know not. I have one rather singular case which will be introduced with a query? being unable to account for the phenomenon.

Patient, Mrs. R., aged 25 years, healthy and robust in appearance, second pregnancy, first child vigorous from birth. Was called to visit her at 10 o'clock A.M.: found her with periodical pains, strongly threatening miscarriage. Had her lie down, keep quiet, etc. Upon examination, thought it advisable to give a full dose of morphine. Left, with instructions to report the case if her sufferings increased. Was called in in about 10 hours; found the womb dilated and ready to discharge its contents. Delivered her of a six months fœtus, which had been dead for a sufficient time to show signs of incipient decomposition. The cord was unusually long and coiled like a cork-screw from end to end. *Query.* Did these coils so impede the circulation as to cause its death? The mother remarked that the life motions had never been so marked as in her former pregnancy.

HYDATID.

This case is introduced to show how abnormal conditions of the womb simulate pregnancy, so as to mislead mothers of experience, also to guard physicians against depending upon other than absolute signs of life in diagnosing pregnancy.

Patient, Mrs. C., aged 30 years, mother of three children. Was requested to visit Mrs. C. by her husband, who stated that she was then in labor. I found her suffering periodical pains: said she had been very sick all day, but had suffered severe pain only a few hours. Stated that her waters had burst one hour since, and that she had literally flooded the place.

This was the evening of April 24th, 1869. Upon inquiry as to whether this was the time she had expected her confinement, she replied that quickening occurred about the middle of December. When questioned whether she had felt life ever since, she replied affirmatively.

During this conversation, by placing my hand upon her abdomen, I found that her child had all run to water; but feeling it my duty to learn whether it was a simple hydatid, or dropsy of an ovum, which might be still there, and, if so, would require removing, I made a vaginal examination. Found the neck firm and resisting, and in such a condition as led me to conclude there had been no considerable dilatation of the os. There was no show of blood. I prescribed an anodyne, with instructions to cease its use upon the cessation of pain. When once quieted, the pain never returned. She was up in due time. The sequel proved it to be an hydatid. I need not add, the family were not a little amazed at the issue of her confinement.

### PROLONGED RETENTION OF FŒTUS.

Sometime in March, 1868, I was engaged to attend Mrs. M. in her confinement, which was expected about the 1st of April. She had been guided in her reckoning not only by the disappearance of her menses, but from her quickening, which occurred about the middle of November. A short time before her expected confinement, she accidentally fell, felt sickened at the result, was alarmed, in view of the probable consequences, but was soon able to be upon her feet, as usual. She afterwards stated to me, that from that time, although there was no discharge of any kind, her size gradually diminished. She was kept in a state of continual suspense, until June 28th, when, after a day's work at the wash tub, she was taken sick and sent for medical aid. Her sickness resulted in the expulsion of a fœtus, much shriveled, but not putrescent, and in size about the average for a six month's gestation. She recovered without any untoward symptom.

### RUPTURE OF UTERUS.

By request, the following very interesting case is furnished by Dr. C. E. Parker, a practitioner of thirty years' experience, late from Beardstown, Ill., and now (having retired from the profession) a citizen of our own city:—

Was called August 9th, 1862, at 2 o'clock P.M., to visit Mrs. R., residing about twelve miles from B. Mr. R. stated she had been in labor during the night, in charge of a neighboring midwife, who at noon reported "a portion of the child born, and something wrong." Arrived at 3½ P.M.; found the patient upon her back, in nearly a sitting position, chest flexed upon the abdomen; lower limbs drawn up. She reported her labor as having been very severe up to 11 o'clock, at which time, after a very violent expulsive effort, "something seemed to give way; all pain ceased, and a strange sickening sensation followed." The hand of the child protruded at 9 o'clock A.M. On manual examination, found a mass of material protruding, consisting of child's arm, a portion of placenta, and a large mass of something which I never met with before. On occular examination, found a large mass of the intestines protruding from the vulva; the partially expelled placenta was easily withdrawn; the hand cautiously introduced, the intestinal coils pushed up before it; found the child in the abdominal cavity; the left foot directly under the liver at its upper portion; the right on the opposite side of the abdomen; cautiously drawing them together, version was gradually produced; the arm slowly returning as the feet were drawn down. The body of the child was extracted readily, but the head brought down a large mass of the intestines, which were with difficulty returned and retained. On minute examination, found a rupture of the uterus on its left posterior lateral portion, the rupture extending through the os tincæ and a portion of the vagina. Allowed her to remain in the same position until all drainage had ceased, then placed her in a horizontal position, hips much elevated, and applied bandages and compresses, calculated to prevent further protrusion of the intestines, and ordered them to be kept saturated with cold water. Gave an opiate, and left her, supposing the injury in itself sufficient to produce death—my manipulations making that result only more certain. The next day Mr. R. called upon me to visit her again. My engagements, the distance, and extreme heat, prevented. Gave him a note to Dr. P., a very worthy and scientific practitioner, residing only three miles from the patient, requesting him to visit her and do all in his power to make her comfortable. On the 12th, received a note from Dr. P., desiring me to meet him at Mr. R's. Found her quite comfortable, and desirous of sitting up. The question for decision, the admissibility of a cathartic. Examined, could detect the fissure in the vagina and os tincæ, but the union seemed firm, but little abdominal soreness, and no pain, except when turning in bed, which the temperature, 98° above, rendered frequently necessary. The cathartic operated kindly, and she was up and about as early as in her six previous labors. Sometime during 1865, she was safely delivered of a child without assistance, save what was rendered by neighboring women. This fact is vouched for by a lady who knew them intimately, and had seen the child repeatedly. I learned this but a short time since, they having removed to a neighboring county some distance, and I having left B. and the profession. In its first phase, I consider the case unique; in its second, I have never heard its parallel.

The following case is kindly furnished by Dr. J. T. Frazer, Howard's Point, Illinois:

In this case there seems to have been a rudimentary brain only, not enough perhaps to render inappropriate the term anencephalus.

"Was called to attend Mrs. R., on March 29, in seventh labor. All former labors natural and without difficulty, except the sixth. Found pelvis and genitalia ample, os uteri but partially dilated, membranes extremely tense and enormously distended. Could not make out presentation, but believed from external configuration that I had a transverse position to deal with. At length the membranes gave way, and I immediately ascertained the presentation to be the upper dorsal spine and posterior of left scapula. Passing two fingers over the shoulders, one on each side of the neck, pressed the breech, etc., towards the left ilium. The head (rudimentary) took the place of the spine and scapula, and revealed the character of the monstrosity. The birth was effected without difficulty."

In describing the fœtus the Doctor states, that although the face was there, the features were badly formed and illy proportioned. For want of space to give minute details, I will only add, that the bones of the base of brain were tolerably well represented, but a complete absence of those forming the vault of the cranium.

Posteriorly there was no brain at all; but extending from one mastoid process to the other, there was a soft liver-like but fibrous substance, and beneath this, representing the cerebellum, was a softer fibrous material of a lighter color. The child did not breathe. The specimen could not be obtained.

The Doctor adds, that this is the fifth case coming under his notice in a practice of 49 years. Three in his own practice (one of which is now in the Museum of the Jefferson Medical College, Penn.), and two inthe practice of a friend.

The following cases, replete with interest, are furnished by Dr. S. R. Crawford, of Monmouth, Illinois:

Case I.—Was called to Mrs. B., in consultation with my friend, Dr. C., June 14th, 1866, and was informed by him that his patient had been suffering severe labor pains for more than 36 hours, without the slightest apparent progress. On making a digital examination, per vaginum, found the os uteri high up in the superior strait, not dilated to any extent whatever, and seemingly not dilatable. The patient, a blonde of a short, stout frame, perfectly developed osseous and muscular systems, 30 years old, and the mother of four children. She informed me that there was no difficulty, and but little delay in her former confinements, that she had no such unbearable pains then as now and in consequence of which she was apprehensive that some insurmountable difficulty stood in the way of successful delivery, and wanted to know how soon I thought she must die. I assured her that nothing was wrong, that she was not now quite ready to be sick, and that there was no good grounds for such uneasiness and apprehension, and advised her physician to administer a good opiate; in response to which she quieted down and slept a few hours, and waked up feeling quite comfortable, and had not the slightest feeling of pain until at the end of seven days from that time she was seized with labor pains, and in a little less than an hour was delivered of a fine, lively female child, weighing between nine and ten pounds; after which she made a speedy recovery, with no peculiar symptoms whatever.

Case II.—May 28th, 1868, was called to see same lady, and found her suffering from the same kind of false pains as before, which kindly yielded to same course of treatment. My patient was comfortable until June 10th, when I was summoned to her, and delivered her in a few minutes of a fine, healthy girl.

I select this case from a considerable number I find in my note book, not for any intrinsic peculiarity in it, but as illustrative of what I consider a rheumatic condition of the gravid womb, developing false pains as gestation approaches completion, resulting in those false alarms, which to the busy practitioner become so contemptible, and which so often bring down maledictions on the heads of accouchers, whose sole crime is good-naturedly and contentedly remaining a day or two awaiting results. A careful examination will generally give a full comprehension of the situation; and in the face of that nefarious phrase, "meddlesome midwifery," when false and spurious pains are suspected, I always give and advise my pupils and young medical friends to give due regard to that suspicion and act accordingly.

Case III.—Mrs. T., a stout German woman, aged 33 years, mother of five children, consulted me Sept. 1st, 1867, concerning herself. She was eight months gone in pregnancy, had noticed nothing unusual until the end of five months, when, on a Sunday, she had gone a few miles into the country in a lumber wagon, and in the evening, after her return, commenced flowing somewhat profusely, and continued, with intervals, in some decreased amount all night, the severity at no time inducing her to call for medical aid. The flowing ceased in the morning, and, with the exception of a feeling of weakness and debility, she felt as well as before. She had no pain at any time. She insisted that she had grown rather smaller than otherwise from that time forward. She followed my advice and gave herself no further trouble about the matter. Two months subsequently I was called, and, after a somewhat protracted and irregular labor, she was delivered of a mass of considerable size, which proved to be the unbroken membranes, containing a small amount of liquor amnii, and a fœtus of as nearly as could be judged of five months development, shriveled and withered in aspect, but not to any extent putrescent. The maternal extremity of the umbillical cord ended in a small, irregular shaped knot, evidently the debris of what was once the placenta, but which was detached at the time of the flowing, five months prior to confinement. Nothing can convince her but that she had been pregnant ten months.

CASE IV.—Was called to Mrs. C., January 30th, 1868, and found her suffering from brisk labor pains. There was a breech presentation, and after a severe labor of three hours duration she gave birth to a living child, with a gaping fissure extending from the nape of the neck to the coccyx, having the appearance of a ragged wound. There was no spinal column to be found. Breathing continued twenty minutes.

CASE V.—Was called August 10th, 1867, to attend Mrs. B. in her sixth labor. A half dozen heroic pains expelled a male child, which, after it was dressed, weighed twenty pounds. Mother and child did well. The little fellow at present is not above the average size. The mother is a large, burly Virginian, and the father, without any considerable amount of obesity, will weigh two hundred pounds. I have been unable to find on record any equal in weight to this child.

N. B.—For remainder of report, see manuscripts (original) of Dr. N. Wright, Chatham, Ill., and B. H. Cheney, Joliet, Ill., appended.

---

The following is kindly furnished by Dr. N. Wright, of Chatham, Illinois:

I was called to Mrs. ——, on the 8th of March last. Was told by her husband that she was in the sixth month of her pregnancy, and that he feared abortion was about to occur. He told me further that she had aborted twice previously. I found her with the pains increasing in severity, recurring regularly, and assuming all the characteristics of ordinary labor pains. Upon examination the mouth of the womb was found dilated to the size of a dollar piece, and a fleshy mass was detected protruding through it. In an about an hour she was delivered of a large mole, weighing, perhaps, four or five pounds. The pains were equal in severity to those of an ordinary labor case, of course excepting those caused by the distention produced by the passage of the child's head. Pure contractile pains are perhaps nearly always equal in severity.

No unusual amount of hemorrhage took place. This mole, in its general outline, bore some resemblance to an afterbirth, being of a fleshy nature. It was studded with little bladder-like bodies, varying in size from a pin's head to a quail's egg. They were of a whitish aspect, the walls were thin, of a fibrous nature, and filled with a thin, serous fluid. An almost infinite number of these cysts existed throughout the mass. This was diagnosed a hydatiform or vessicular mole, or, vulgarly, a false conception—the vessicles resulting from a transformation of the cells in the villi of the chorion, as maintained by Paget and others—this disease, or degeneration of the placenta, producing the death of the embryo, by deranging and cutting off its nutrition. Dr. Graily Hewitt, on the other hand, reverses this order, and contends that the death of the embryo, from some causes, brings about this peculiar change in the placenta.

